# Risk-Profile and Feature Selection Comparison in Diabetic Retinopathy

**DOI:** 10.3390/jpm11121327

**Published:** 2021-12-08

**Authors:** Valeria Maeda-Gutiérrez, Carlos E. Galván-Tejada, Miguel Cruz, Jorge I. Galván-Tejada, Hamurabi Gamboa-Rosales, Alejandra García-Hernández, Huizilopoztli Luna-García, Irma Gonzalez-Curiel, Mónica Martínez-Acuña

**Affiliations:** 1 Unidad Académica de Ingeniería Eléctrica, Universidad Autónoma de Zacatecas, Jardín Juarez 147, Centro 98000, Mexico; valeria.maeda@uaz.edu.mx (V.M.-G.); gatejo@uaz.edu.mx (J.I.G.-T.); hamurabigr@uaz.edu.mx (H.G.-R.); alegarcia@uaz.edu.mx (A.G.-H.); hlugar@uaz.edu.mx (H.L.-G.); 2 Unidad de Investigación Médica en Bioquímica, Centro Médico Nacional Siglo XXI, Hospital de Especialidades, Instituto Mexicano del Seguro Social, Mexico City, Av. Cuauhtémoc 330, Col. Doctores, Del. Cuauhtémoc, Ciudad de Mexico 06720, Mexico; miguel.cruzlo@imss.gob.mx; 3 Unidad Académica de Ciencias Químicas, Universidad Autónoma de Zacatecas, Jardín Juarez 147, Centro 98000, Mexico; irmacuriel@uaz.edu.mx (I.G.-C.); monicaimeldamtza@uaz.edu.mx (M.M.-A.)

**Keywords:** diabetic retinopathy, feature selection, random forest, risk factors

## Abstract

One of the main microvascular complications presented in the Mexican population is diabetic retinopathy which affects 27.50% of individuals with type 2 diabetes. Therefore, the purpose of this study is to construct a predictive model to find out the risk factors of this complication. The dataset contained a total of 298 subjects, including clinical and paraclinical features. An analysis was constructed using machine learning techniques including Boruta as a feature selection method, and random forest as classification algorithm. The model was evaluated through a statistical test based on sensitivity, specificity, area under the curve (AUC), and receiving operating characteristic (ROC) curve. The results present significant values obtained by the model obtaining 69% of AUC. Moreover, a risk evaluation was incorporated to evaluate the impact of the predictors. The proposed method identifies creatinine, lipid treatment, glomerular filtration rate, waist hip ratio, total cholesterol, and high density lipoprotein as risk factors in Mexican subjects. The odds ratio increases by 3.5916 times for control patients which have high levels of cholesterol. It is possible to conclude that this proposed methodology is a preliminary computer-aided diagnosis tool for clinical decision-helping to identify the diagnosis of DR.

## 1. Introduction

Diabetes Mellitus (DM) is a metabolic disorder characterized by hyperglycemia, resulting from the inability of the pancreas to produce enough insulin [1].

Distinguished as a global health emergency, it affects more than 463 million people worldwide; and it is expected to exceed 578 million by 2030 and 700 by 2045, thus becoming the seventh leading cause of death in 2030 [2]. Another alarming aspect is the high percentage of people with undiagnosed DM, which currently exceeds 50%, being in most cases type 2 diabetes (T2D) [2]. T2D represents 90–95% of DM cases worldwide; results from a pancreatic β cell dysfunction combined by insulin resistance. Furthermore, it can lead to an increased risk of complications which impact people’s quality of life, their finances, and generate an economic burden for the health system. It is important to point out that T2D is prevalent in Latin Americans due to a combination of genetic and lifestyle risk factors [3]. For the specific case of Mexico, the annual cost generated by DM is 17 billion USD; it is also in the list of the top ten countries with undiagnosed diabetics, where 4.9 million people have DM and do not know it. On the other hand, it is estimated that the prevalence of DM by 2030 will be 17.2 million people and consequently it will increase by 5% in 2045 [2].

One of the main chronic complications of DM in Mexico is diabetic retinopathy (DR), which occurs when there is excess glucose in the bloodstream that damages the blood vessels in the retina, and this is the leading cause of blindness in working-age adults (20–65 years) [4], being frequent in up to 40% of all those affected by DM. In addition, it ranks third in direct costs, which include diagnostic and treatment procedures [5,6]. There are five stages of DR. The first is “no apparent DR”; the second is “mild non-proliferative DR” (NPDR) which is distinguished by the presence of some microaneurysms; “moderate NPDR” is the third, characterized by the presence of microaneurysms, and intraretinal hemorrhages. The fourth stage is “severe NPDR”; and the last stage is “proliferative DR”. This presents neovascularization of the disc, retina, iris, hemorrhage, of the angle, vitreous hemorrhage, or tractional retinal detachment [7]. There are many treatments for DR, an eye examination by an ophthalmologist or optometrist is important. In addition, high-quality fundus photographs can detect DR and serve as a tool. Several studies, taking advantage of digital computing and artificial intelligence advances, have implemented deep neural networks for automated detection of DR in retinal fundus photographs. The implementation of convolutional neural networks (CNNs) for the recognition task of DR stages have been successful in the medical field; some authors [8,9,10,11] used CNN architectures such as AlexNet, GoogleNet, and VGGNet, where finally, significant results were obtained.

Some research has focused on identifying risk factors for DR; Semeraro et al. [12], quantify the individual risk for DR in T2D patients by using c-statistic, area under the curve, and the Gonen and Heller concordance probability estimate (CPE) for the Cox proportional hazard model. The external validation showed 0.767 and 0.697 for C-index and CPE, respectively. The risk factors associated with DR were duration of DM, glycosylated hemoglobin (HbA1C), systolic blood pressure, male gender, albuminuria, and DM therapy. Another approach conducted by Alfian et al. [13] proposed a Deep Neural Network (DNN) combined with Recursive Feature Elimination (RFE) to provide early prediction of DR using known risk factors. There were 13 attributes found, but the DM duration, subject’s fasting blood sugar level (FBS), high density lipoprotein (HDL), age, and HbA1C were the significant risk factors. Then, the authors compared the outcomes with several machine learning models: k-Nearest Neighbor (KNN), C4.5 Decision Tree (DT), Support Vector Machine (SVM), Naïve Bayes (NB), Random Forest(RF), and their proposed model (DNN-RFE); Finally, the area under the curve for each model was 0.70 (KNN), 0.73 (DT), 0.71 (SVM), 0.71 (NB), 0.68 (RF), and 0.80 (DNN-RFE). The investigation done by Wang et al. [14], focused on identifying a set of laboratory tests that were the most important for DR prediction. In the analysis the predictive model achieved 0.85 area under the curve on the derivation cohort and 0.77 on the validation. The essential predictors for DR were: creatinine, HbA1C, neuropathy, DM duration, age, nephropathy, WBC, hematocrit, and sodium.

As shown, many studies describe the implementation of different models of artificial intelligence for the classification of the different stages of DR, and others to find risk factors that are associated with this disease, but it remains unknown which factors are more associated. The main contribution of this study focuses on identifying possible predictors of DR. Thus, feature selection techniques are used to investigate the discriminative features for DR, and to finally evaluate each attribute with the aim of knowing which of them are predominant in the progress and the development of the DR.

## 2. Materials and Methods

**Figure 1:**
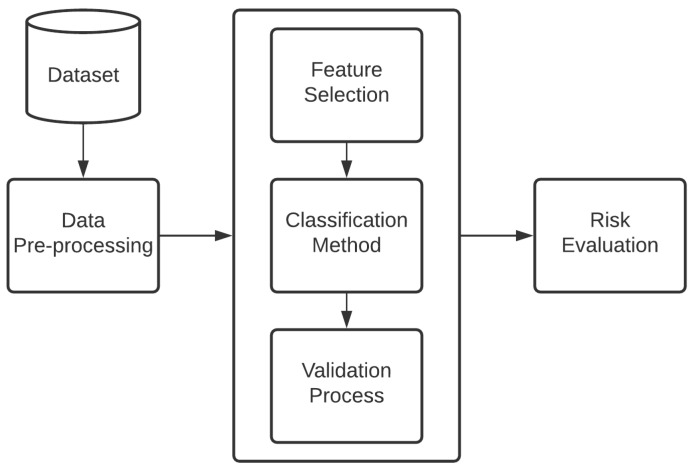
Flowchart representing the proposed methodology.

In Figure 1, the entire procedure is divided into six basic steps: data acquisition, data pre-processing, feature selection, data classification, data evaluation, and the risk evaluation for each feature.

### 2.1. Data Description

The dataset for this study was provided by the *Unidad de Investigación Médica en Bioquímica, Centro Médico Nacional Siglo XXI, Instituto Mexicano del Seguro Social* (IMSS). The Mexican patients signed an informed consent letter and the protocol meets the Helsinki criteria which were approved by the Ethics Committee of IMSS under the number R-2011-785-018. Table 1 shows basic information, laboratory indicators, and medical treatment. These data were extracted for each subject and used for the analysis.

The dataset is comprised of 32 input variables, and one categorical output. For this study, there were a total of 298 participants. The case group comprises 149 T2D patients with DR, and the control group comprised 149 T2D patients without DR. All subjects aged between 30 and 84 years. There were 173 female and 125 male samples, accounting for 58.08% and 41.94%, respectively with the portion of female samples being high. Table 2 and Table 3 show the independent variable features, indicating values as mean, standard deviation, and percentages (%). Table 4 displays the categorical attributes (0-No, 1-Yes), which “1” indicates that the subject received medical treatment, and “0” means that the subject is off medical treatment, and finally, Table 5 shows the *p*-values aiming to compare cases and controls.

### 2.2. Data Pre-Processing

For the pre-processing stage, the first step consisted of manually removing those input features (ID, neuropathy, and nephropathy cases) from the original dataset that are not relevant for this study. Furthermore, the remaining input features presented some missing values (GFR, SBP, DBP, USBP, UDBP) represented as NA were imputed with the value calculated mean of the non-missing observations.

### 2.3. Feature Selection

Boruta is a wrapper method based on Random Forest (RF). This algorithm was applied to select all the possible predictors of DR. In short, Boruta expands the data set by creating “shadow” variables for each input variable; these added attributes are shuffled to eliminate their correlations with the response; an RF classifier that uses all the features is executed, both the original and the shadow ones, and the Z scores are collected; the Z score is the measure with which the importance of each attribute is determined by assigning three possible labels to each feature; rejected, tentative, and confirmed, then the maximum Z score among the shadow attributes (MZSA) is found to assign a value to each attribute that scored better than MZSA. In addition, for an attribute with indeterminate importance, a two-sided test of equality is performed with the MZSA, where the attributes that have significantly less importance than MZSA are rejected. Finally, those that have significantly greater importance are considered [15].

### 2.4. Classification Method

The supervised machine learning algorithms can learn features of the target classes from the training set and are capable of identifying these learned features in the testing set. An RF approach was implemented, in order to develop a general model that identifies a diabetic patient that also has DR or a diabetic patient that does not have DR.

#### Random Forest

Developed by Breiman et al. [16], RF is a popular machine learning algorithm. This technique is a combination of decision trees (DT); each DT provides a vote; then, the results are acquired by adding the vote from different decision trees to decide the final class, according to the principle of majority.

The specific steps are described as follows:

- First, the dataset $D_{1}$ having mxn is given; the samples are randomly selected using the bootstrap method, which is used to create a new dataset $D_{2}$.
- Next, the RF algorithm is trained to generate a DT for every sample; subsequently, the unbiased error is estimated.
- In this step, the final prediction is calculated based on the number of votes of the DT.
- Finally, the samples that have not been selected in the training process can be used in the testing process to evaluate the performance of the classifier.

### 2.5. Validation Process

To evaluate the performance and validate the classification model obtained, the confusion matrix, the ROC curve, and the AUC value are measured. Two possible outputs in a classification problem are 0 and 1; they can be represented within a confusion matrix. This table contains True Positives (TP), True Negatives (TN), False Positives (FP), and False Negatives (FN). As well as the diagonal which represents the observations that are correctly classified. On the contrary, those that remain outside the diagonal correspond to the observations that were misclassified.

It is possible to obtain other important metrics to evaluate the quality of the model by analyzing how well it performs on test data. However, in this work it was used to calculate the following metrics: sensitivity, specificity, and Area Under the Curve (AUC).

Sensitivity (recall), represents the portion of true positives; in other words, the ability to identify those with a DR condition, shown in Equation (1).

(1) $$Sensitivity=\frac{TP}{TP+FN}$$

Specificity, measures the portion of true negatives that are correctly identified; e.g.,: the ability to identify those without a DR condition, shown in Equation (2).

(2) $$Specificity=\frac{TN}{TN+FP}$$

The ROC curve plots the relationship between sensitivity (*y*-axis) and specificity (*x*-axis), providing important information about the performance of a binary classifier. This analysis can discriminate between two classes: with DR (diseased) or without DR (non-diseased). Another significant metric complemented with the ROC curve is AUC, which gives the probability that a random sample would be correctly classified by the model. In practice, the AUC value ranges from 0.5 to 1, wherein in the case of perfect class separation (performance), the AUC will be the higher value. [17,18]

### 2.6. Risk Evaluation

To evaluate the effect of the predictor variables obtained by boruta, both the relative risk and odds ratio were evaluated in our study population.

The *Relative Risk* (RR) or risk ratio is a measure of association between the exposure and the control group or a particular outcome; it is calculated by the Equation (3).

(3) $$RelativeRisk=\frac{RiskofExposureGroup}{RiskofUnexposedGroup}$$

Assuming the causal effect between the two groups: cases and controls, the values of RR can be interpreted as follows:

- RR = 1.0, indicates no difference between the two groups.
- RR < 1.0, indicates a negative association; the risk of the microvascular complication (DR) decrease when the factor risk is present.
- RR > 1.0, indicates a positive association; the risk of the microvascular complication (DR) increase when the risk factor is present.

The *Odds Ratio* (OR) defined by the odds of disease in the exposed group divided by the odds of disease in the unexposed group; are often interpreted as the RR.

All the methodology was developed in R (version 4.0.3) [19]. The libraries used were Boruta (version 7.0) [15], caret (version 6.0-86) [20], MLeval (version 0.3) [21], fsmb (version 4.0.5) [22], and epitools (version 4.0.5) [23].

## 3. Experiments and Results

The dataset used in this study was extracted from the *Centro Médico Nacional Siglo XXI*, IMSS. A total of 298 subjects were divided into two classes: cases (n = 149) and controls (n = 149), where the cases present T2D with DR, and the control group are the subjects with T2D. There were 32 input variables and one outcome variable, that were used for the development of the model. The input variables were: basic information, biochemical indicators, and the medical treatment.

**Figure 2:**
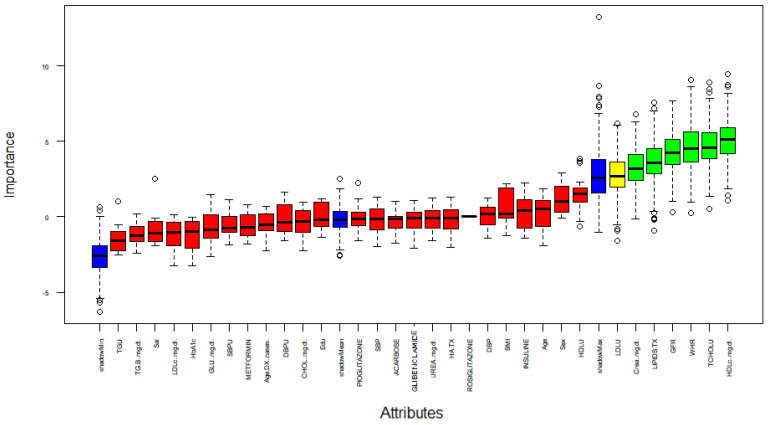
Feature selection process according by Boruta.

First, a feature selection was performed using Boruta; this method captures all the relevant features for the classification in terms of importance. Figure 2 indicates the importance on y−axis of the analyzed attributes (x−axis). The blue box plots correspond to the shadow attributes. There are only three: shadow minimum, average, and maximum Z score; the red box-plots represent the irrelevant features; the yellow box-plots correspond to the tentative attributes; and the green box-plots correspond to the confirmed features.

Aiming to obtain the relevant features, the Boruta algorithm performed 499 iterations. The selection results are summarized in Table 6. In this case, out of 32 features, 25 are rejected, one is tentative, and six are confirmed. In addition, the Norm Hits are the number of the RF runs in which this feature was more important than the shadow one.

Based on the result: Creatinine, Lipids TX, GFR, WHR, TCHOLU, and HDL are possible predictors of DR. On the other hand, these confirmed attributes are relevant for the classification of DR.

The selected attributes serve as the input variables for the RF algorithm. Basically, RF creates numerous DT’s that produce more accurate classifications. RF needs some additional hyper-parameters that should be considered. The type of RF is “classification”, and the number of trees (ntree) is 500. To evaluate the performance of the model, a K-Fold Cross-Validation (KCV) was performed, and calculated its sensitivity, specificity, confusion matrix, ROC curve, and AUC value. In this study, 10-Fold CV was implemented, it consists in splitting the dataset in K independent subsets, in this case were 10, in turn nine of these subsets are used to train the RF classifier, while the remaining one is used to evaluate the error.

**Figure 3:**
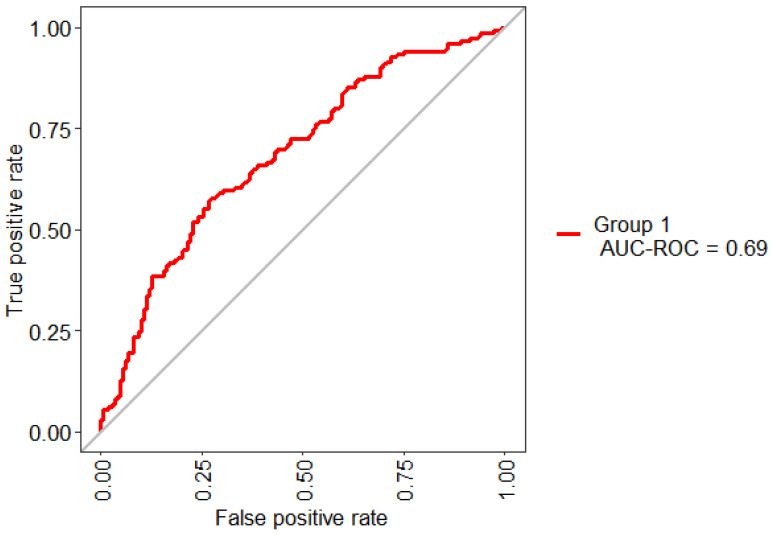
ROC curve obtained for the model based on the key features identified by Boruta.

Figure 3 shows the statistical model performance obtained by implementing Boruta + RF with 10-Folds CV. This method presented an AUC of 69% and an Out-Of-Bag (OOB) error of 37.92%.

According to the Fawcett criterion, the interpretation of the AUC values is as follows:

- Bad test = (0.5, 0.6)
- Regular test = (0.6, 0.75)
- Good test = (0.75, 0.9)
- Very good test = (0.9, 0.97)
- Excellent test = (0.97, 1) [24]

These values were used to interpret the performance of the model.

Table 7 shows the detail performance of the RF model. This model obtained an AUC value of 69% indicating that the model has the ability to diagnose patients with DR. In medical diagnosis, sensitivity provides the portion of positive instances that were correctly classified, and the specificity, the portion of negative instances that were correctly classified. The results indicate that the RF model is capable of classifying both cases: subjects with T2D and DR; otherwise, subjects with T2D without DR.

The confusion matrix of the RF model is presented in Table 8. Depending on the results it is possible to evaluate the classifier and determine which classes are highlighted. The off-diagonal corresponds to the observations that were incorrectly classified, and the correct predictions are located in the diagonal.

In order to evaluate those features that imply the development of DR, both groups were divided by condition. Regarding the controls, 53 of them have lipid treatment; in relation to GFR, 59 present levels below 90 mL/min; with respect to the WHR index, 56 of them showed higher values compared to the normal index; 69 of type 2 diabetics have high levels of total cholesterol; likewise, in 133 subjects low levels of HDL were found; and finally, in regards to creatinine, 24 controls have values out of range, as shown in Table 9.

With the above, the RR associated with having DR was calculated, as shown in Table 10. The results show a statistically significant positive association between DR and the predictors found by Boruta, in which there is a risk of suffering from DR if a control subject has one of these factors.

In Table 11 the odds ratio was calculated; which is a measure of association that estimates how the presence of certain factors influences the development of a condition. In the present study, the variables confirmed by Boruta were analyzed and it was found that the risk increases if any of these factors are present. For example, the odds of developing DR increases 2.0575 times if the subject presents levels of GFR below 90 mL/min; regarding total cholesterol, this increases the odds of suffering DR by 3.5916 times.

## 4. Discussion and Conclusions

In this section, the discussion and conclusion of the results obtained are presented. Initially, the Mexican dataset contained 47 input features. Nevertheless, a pre-processing stage was performed, removing all the variables that are not relevant to this study. Thus, the final dataset was comprised of 32 features listed in Table 1. The total number of patients used is 298, of which 149 (cases) correspond to diabetic patients with DR, and the remaining 149 (controls) correspond to diabetic patients.

This work employed machine learning algorithms to classify DR cases among T2D patients and provided possible risk factors. Feature selection has been used in various domains [25,26] to improve the model prediction. The main idea of this method is to choose important features that are essential for the discovery of new knowledge [27]; a small subset of the significant features subsequently reduce the computation time. Boruta was implemented for this task, the wrapper algorithm determines the importance for each feature; the benefits of this algorithm is that it considers all attributes that are related to the output, and it considers multi-variable relationships where it can also explore interactions between variables [28]; the risk factors for DR were identified with the most important predictors being lipids treatment, glomerular filtration rate, waist hip-ratio, total cholesterol, high density lipoprotein, and creatinine. These six features comprise the new dataset. RF has been successfully used in many healthcare approaches, such as the development of a model to predict the severity of a COVID-19 case and the possible outcome, recovery, or death [29], as well as for the prediction of anxiety, depression, and stress [30], the diagnosis and prognostic for breast cancer [31], and the modelling for the diagnosis of pediatric pneumonia from chest X-ray images [32], among others. An RF model was built to classify the two different classes of subjects; then, an approach of 10-Fold CV was applied. Once the model has been built, it is important to evaluate the performance; the prediction of the RF model was based on different metrics used in clinical areas; sensitivity provides the portion of positive instances that were classified correctly, specificity is the portion of negative instances that were correctly classified, the ROC curve is the plot of the sensitivity versus 1-specificity, this curve includes all the possible decision thresholds from a diagnostic test result; and the AUC value demonstrates the performance of the model to detect or classify if a patient has the disease. An ideal performance has an AUC of 1; conversely, a value < 0.5 is considered to have a reasonably discriminating ability. Table 12 presents a comparison of the different approaches used; in the first model, the 32 features were evaluated, while the second model uses Boruta, which is in charge of determining the importance of each feature. The prediction of both models is in an acceptable performance according to the Fawcett criterion. The general model, which submitted 32 attributes, had lower results in terms of sensitivity, specificity, and AUC. On the other hand, the model with only six features obtained the highest results, and it demonstrated the effectiveness of implementing feature selection.

To compare the RF results with other machine learning algorithms, using the six characteristics obtained by Boruta, Table 13 shows the sensitivity, specificity, and AUC values of RF, Support Vector Machine (SVM), and Logistic Regression (LR) models. It can be concluded that in terms of AUC values and specificity, RF achieved the best performance.

There are many DR risk factors cited in the literature such as duration of DM, high blood glucose, poor blood glucose control, high blood pressure, dyslipidemia/high cholesterol, nephropathy, obesity, insulin treatment, smoking, ethnicity, and age [4,33,34,35,36,37]. The study has demonstrated the relationship between DR and Lipids TX + GFR + WHR + TCHOLU + HDL + Creatinine. Initially, the research of Klein et al. [38], demonstrated that HDL and the cholesterol levels were not related to the severity of the DR, but were a significant factor in describing the severity of retinal hard exudate. Similarly, the study of Chang and Wu [39] confirmed the association of total cholesterol with the presence of hard exudates in patients with DR. Additionally, Müller et al. [40], investigated the association between DM and lipid-related biomarkers and DR in hemodialysis patients, their results showed that DR was associated with HDL. Besides, Van et al. [41], prove that the prevalence of DR was positively associated with cholesterol. It is important to mention that HDL is directly related to macrovascular complications of T2D. Another serious complication of T2D is diabetic nephropathy (DN), which is more common in subjects with DR; and the severity of DN increases with DR. Zang et al. [42] conclude that serum creatinine and GFR are associated with the presence and severity of DR. Likewise, El Haddad et al. [43], study the prevalence of DR, demonstrating the significant factors related to the occurrence of DR; and it was found that the duration of DM was the only risk factor associated with mild NPR, while high levels of serum creatinine, cholesterol, and triglycerides were significantly associated with the occurrence of proliferative retinopathy. On the other hand, the obesity in individuals with DM is associated with DR, specially the abdominal obesity and the increased WHR were correlated with the DR but not with the severity [44]. Klein et al. [45], suggest that WHR and index of regional body fat distribution is associated with DR. On the other hand with the aim of knowing the impact of each risk factor, a risk evaluation was done. The RR and OR are the most ubiquitous measures of association and risk quantification in medical research [46]. With regard to Lipids TX, the dataset only mentions whether the subjects are under medical lipid treatment. It should be noted that if the patient is undergoing the treatment and still has high cholesterol levels he or she is prone to developing DR. The results showed a positive statistical association, the development of DR increase 3.5916 times if the control patient presents high levels of cholesterol, which is a strong indicator of DR.

In conclusion, this paper focuses on identifying the predictors of DR based on a methodology contained in five main stages. For the feature selection process Boruta has confirmed six attributes; then, a classification stage was done using RF; afterward, the performance measures were calculated. Finally, the model achieved 69% of AUC, demonstrating the effectiveness of classifying patients with DR. A timely diagnosis, continuous medical care, and effective medical treatments are necessary; there is a need to identify risk factors related with DR which may facilitate a better understanding of it. These results can be used to facilitate the development of a preliminary tool that can be useful to support specialists in the diagnosis of DR, and may improve their decision-making in the management of this diabetic complication. For future work, it would be interesting to include a dataset with DR images of Mexican subjects which probably allows increasing the performance.

## Figures and Tables

**Table 1:** Features gathered from the Mexican dataset.

Feature Category	Feature
Basic information	EDU, SAL, SEX, AGE, AGE DX, WHR, BMI, SBP, DBP USBP, UDBP
Biochemical indicators	GLU, UREA, CRE, CHOL, HDL, LDL, TG, TCHOLU, UHDL, ULDL, UTG, HBA1C, GFR
Additional information	GB, MF, PG, RG, AB, INS, LIPIDS TX, HA-TX
Output	DIABETIC RETINOPATHY

Note: EDU(Education, studies concluded by the subject); SAL (Salary, monthly income); SEX (subject sex); AGE (subject age); AGE DX (age at diagnosis of diabetes); WHR (waist hip ratio); BMI (body mass index); SBP (systolic blood pressure); DBP (diastolic blood pressure); USBP (systolic blood pressure uncorrected by treatment); UDBP (diastolic blood pressure uncorrected by treatment); GLU (blood glucose levels); UREA (waste product resulting from the breakdown of protein in the subject body); CRE (waste product produced by muscles as part of regular activity); CHOL (fat-like substance that is found in all cells of the subject body); HDL (high density lipoprotein); LDL (low density lipoprotein); TG (type of fat found in the subject body); TCHOLU (total cholesterol uncorrected); UHDL (high density lipoprotein uncorrected by treatment); ULDL (low density lipoprotein uncorrected by treatment); UTG (triglycerides uncorrected by treatment); HBA1C (glycated hemoglobin); GFR (glomerular filtration rate); GB (drug treatment); MF (drug treatment); PG (drug treatment); RG (drug treatment); AB (drug treatment); INS (drug treatment); LIPIDS TX (lipids treatment); HA-TX (hypertension treatment).

**Table 2:** General characteristics of all the subjects included.

Feature	Cases (n = 149)	Controls (n = 149)
Education, *p*-value = <2.2 × $10^{-16}$		
Elementary school Secondary school Technical school High school Professional Postgraduate	25 (16.77%) 33 (22.14%) 24 (16.10%) 30 (20.13%) 34 (22.81%) 3 (2.01)	39 (26.17%) 32 (21.47%) 17 (11.40%) 12 (8.05%) 44 (29.53%) 5 (3.35%)
Salary, *p*-value = <2.2 × $10^{-16}$		
Less than $2000.00 Between $2000.00 and $5000.00 More than $5000.00	35 (23.48%) 66 (44.29%) 48 (32.21%)	37 (24.83%) 63 (42.28%) 49 (32.88%)
Sex, *p*-value = <2.2 × $10^{-16}$		
Female Male	75 (50.33%) 74 (49.66%)	98 (65.77%) 51 (34.22%)
Age (years)	57 ± 9.96	55 ± 9.42
Age DX (years)	44.09 ± 7.55	45 ± 7.06
WHR (cm/cm)	0.94 ± 0.07	0.91 ± 0.07
BMI (kg/m2)	29.55 ± 5.00	30.02 ± 5.07
SBP (mmHg)	125 ± 15.88	123.41 ± 14.95
DBP (mmHg)	81.93 ± 11.09	83.82 ± 11.11
USBP (mmHg)	122.32 ± 15.01	120.06 ± 13.92
UDBP (mmHg)	80.27 ± 10.65	82.14 ± 10.62

**Table 3:** Biochemical indicators of all the subjects included.

Feature	Cases (n = 149)	Controls (n = 149)
Glucose (mg/dL)	162.02 ± 70.44	165.51 ± 68.67
Urea (mg/dL)	38.14 ± 22.83	34.45 ± 16.91
Creatinine (mg/dL)	0.96 ± 0.59	0.84 ± 0.28
Cholesterol (mg/dL)	209.54 ± 46.38	219.55 ± 52.56
HDL (mg/dL)	39.24 ± 12.88	43.95 ± 13.34
LDL (mg/dL)	148.50 ± 39.13	154.86 ± 41.55
Triglycerides (mg/dL)	231.49 ± 157.77	214.16 ± 112.94
TCHOLU (mg/dL)	184.71 ± 40.29	201.58 ± 46.47
UHDL (mg/dL)	41.71 ± 12.40	45.64 ± 13.37
ULDL (mg/dL)	125.43 ± 33.15	138.04 ± 34.55
UTG (mg/dL)	208.07 ± 152.53	198.26 ± 111.44
HBA1C (mmol/L)	7.74 ± 3.15	7.47 ± 2.58
GFR (mL/min)	98.31 ± 41.27	101.88 ± 33.65

**Table 4:** Additional information of all the subjects included.

Feature	Cases (n = 149)	Controls (n = 149)
Glibenclamide		
0 1	74 (49.66%) 75 (50.33%)	87 (58.38%) 62 (41.61%)
Metformin		
0 1	35 (23.48%) 114 (76.51%)	32 (21.41%) 117 (78.52%)
Pioglitazone		
0 1	147 (98.65%) 2 (1.34%)	144 (96.64%) 5 (3.35%)
Rosiglitazone		
0 1	149 (100%) -	149 (100%) -
Acarbose		
0 1	147 (98.65%) 2 (1.34%)	148 (99.32%) 1 (0.67%)
Insuline		
0 1	103 (69.12%) 46 (30.87%)	116 (77.85%) 33 (22.14%)
HA.TX		
0 1	100 (67.11%) 49 (32.88%)	99 (66.44%) 50 (33.55%)
Lipids TX		
0 1	75 (50.33%) 74 (49.33%)	96 (64.42%) 53 (35.57%)

**Table 5:** *p*-value of each feature.

*p*-Value		
**Features**	**Cases**	**Controls**
Education	<2.2 × $10^{-16}$	<2.2 × $10^{-16}$
Salary	<2.2 × $10^{-16}$	<2.2 × $10^{-16}$
Sex	<2.2 × $10^{-16}$	<2.2 × $10^{-16}$
Age	<2.2 × $10^{-16}$	<2.2 × $10^{-16}$
Age DX	<2.2 × $10^{-16}$	<2.2 × $10^{-16}$
WHR	4.054 × $10^{-16}$	<2.2 × $10^{-16}$
BMI	<2.2 × $10^{-16}$	<2.2 × $10^{-16}$
SBP	<2.2 × $10^{-16}$	<2.2 × $10^{-16}$
DBP	<2.2 × $10^{-16}$	<2.2 × $10^{-16}$
USBP	<2.2 × $10^{-16}$	<2.2 × $10^{-16}$
UDBP	<2.2 × $10^{-16}$	<2.2 × $10^{-16}$
Glucose	<2.2 × $10^{-16}$	<2.2 × $10^{-16}$
Urea	<2.2 × $10^{-16}$	<2.2 × $10^{-16}$
Creatinine	0.515	<2.2 × $10^{-16}$
Cholesterol	<2.2 × $10^{-16}$	<2.2 × $10^{-16}$
HDL	<2.2 × $10^{-16}$	<2.2 × $10^{-16}$
LDL	<2.2 × $10^{-16}$	<2.2 × $10^{-16}$
Triglycerides	<2.2 × $10^{-16}$	<2.2 × $10^{-16}$
TCHOLU	<2.2 × $10^{-16}$	<2.2 × $10^{-16}$
UHDL	<2.2 × $10^{-16}$	<2.2 × $10^{-16}$
ULDL	<2.2 × $10^{-16}$	<2.2 × $10^{-16}$
UTG	<2.2 × $10^{-16}$	<2.2 × $10^{-16}$
HBA1C	<2.2 × $10^{-16}$	<2.2 × $10^{-16}$
GFR	<2.2 × $10^{-16}$	<2.2 × $10^{-16}$
Glibenclamide	<2.2 × $10^{-16}$	<2.2 × $10^{-16}$
Metformin	3.87 × $10^{-10}$	<2.2 × $10^{-16}$
Pioglitazone	<2.2 × $10^{-16}$	0.02484
Rosiglitazone	NA	NA
Acarbose	<2.2 × $10^{-16}$	0.3189
Insuline	<2.2 × $10^{-16}$	1.224 × $10^{-09}$
HA.TX	<2.2 × $10^{-16}$	8.107 × $10^{-15}$
Lipids TX	<2.2 × $10^{-16}$	8.086 × $10^{-16}$

**Table 6:** Feature selection results—Confirmed attributes.

No.	Attribute	Feature Selection-Boruta
		Norm Hits
1	Creatinine	0.6355
2	Lipids TX	0.7054
3	GFR	0.7615
4	WHR	0.8316
5	TCHOLU	0.8537
6	HDL	0.8777

**Table 7:** Performance metrics of RF classifier.

Classifier	Sensitivity	Specificity	AUC
Random Forest	0.6422	0.6169	0.69

**Table 8:** Confusion matrix of the RF classifier.

		Reference		
		**0**	**1**	**Class. Error**
Prediction	0	91	58	0.3892
	1	55	94	0.3691

**Table 9:** Participants groups divided by condition.

Lipids Profile	Cases	Controls	Total
Lipids TX	74	53	127
GFR	66	59	125
WHR	72	56	128
TCHOLU	45	69	114
HDL	139	133	272
Creatinine	37	24	61
Total	433	394	827

**Table 10:** Risk Ratio with 95% C.I.

Lipids Profile	Estimate	Lower	Upper
Lipids TX	1	NA	NA
GFR	1.1310	0.8575	1.4916
WHR	1.0483	0.7889	1.3930
TCHOLU	1.4503	1.1257	1.8686
HDL	1.1716	0.9228	1.4876
Creatinine	0.9427	0.6490	1.3693

**Table 11:** Odds Ratio with 95% C.I.

Lipids Profile	Estimate	Lower	Upper
Lipids TX	1	NA	NA
GFR	1.2466	0.7571	2.0575
WHR	1.0854	0.6596	1.7877
TCHOLU	2.1316	1.2759	3.5916
HDL	1.3342	0.8726	2.0494
Creatinine	0.9073	0.4815	1.6910

**Table 12:** Results for DR classification.

	Sensitivity	Specificity	AUC
32 features + RF	0.5709	0.5634	0.61
Boruta approach + RF	0.6422	0.6169	0.69

**Table 13:** Comparison of machine learning models.

Classifier	Sensitivity	Specificity	AUC
RF	0.6422	0.6169	0.69
LR	0.6714	0.5858	0.68
SVM	0.6647	0.5882	0.67

## Data Availability

Not applicable.
